# Supplementary material for: Who is who in necromass formation and stabilization in soil? The role of fungi and bacteria as complementary players of biogeochemical functioning
Source: ISME Commun. 2025 Nov 7;5(1):ycaf186. doi: 10.1093/ismeco/ycaf186 (PMC12642871; doi:10.1093/ismeco/ycaf186)
Supplement: Supplementary_Material_Lepori_et_al_ycaf186 [file supplementary_material_lepori_et_al_ycaf186.docx]

# Supplementary Material

**Supplementary Table 1.** Composition of different communities. The single microbes were ordered from the catalogue of the DSMZ collection. (<https://www.dsmz.de/collection/catalogue/microorganisms/catalogue>).

| **Community composition** | **Microbial inocula** |
| --- | --- |
| BF_complex_ | Natural soil sample |
| BF_simple_ | *Streptomyces sp.* (DSM 687), *Microvirgula aerodenitrificans* (DSM 736), and *Trichoderma koningii* (DSM 63059) |
| B_only_ | *Streptomyces sp.* and *Microvirgula aerodenitrificans* |
| F_only_ | *Trichoderma koningii* |


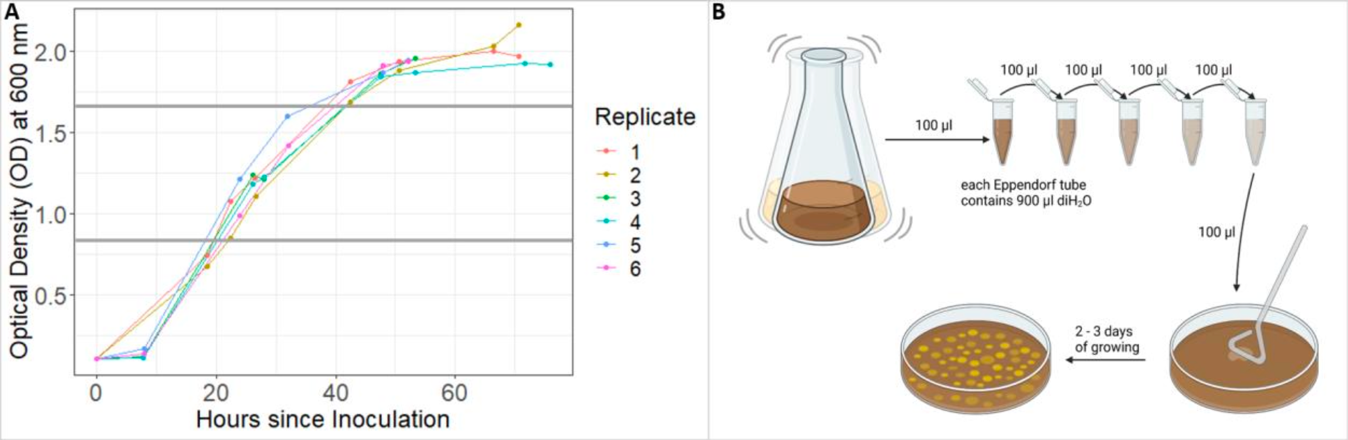


Supplementary Figure 1. Optical density measurements of Microvirgula aerodenitrificans (DSM 736) during strain growth (A) and colony forming units (CFU) approach (B) used to determine inoculum volume to achieve the targeted cell abundance. CFU counting were performed at two points during exponential growth (indicated by the grey horizontal lines). For this, serial 10-fold dilutions were performed until reaching a dilution of 10^-5^ and plated on TSA plates. The plates were replicated 6 times and the CFUs were counted 2 or 3 days after plating.

| 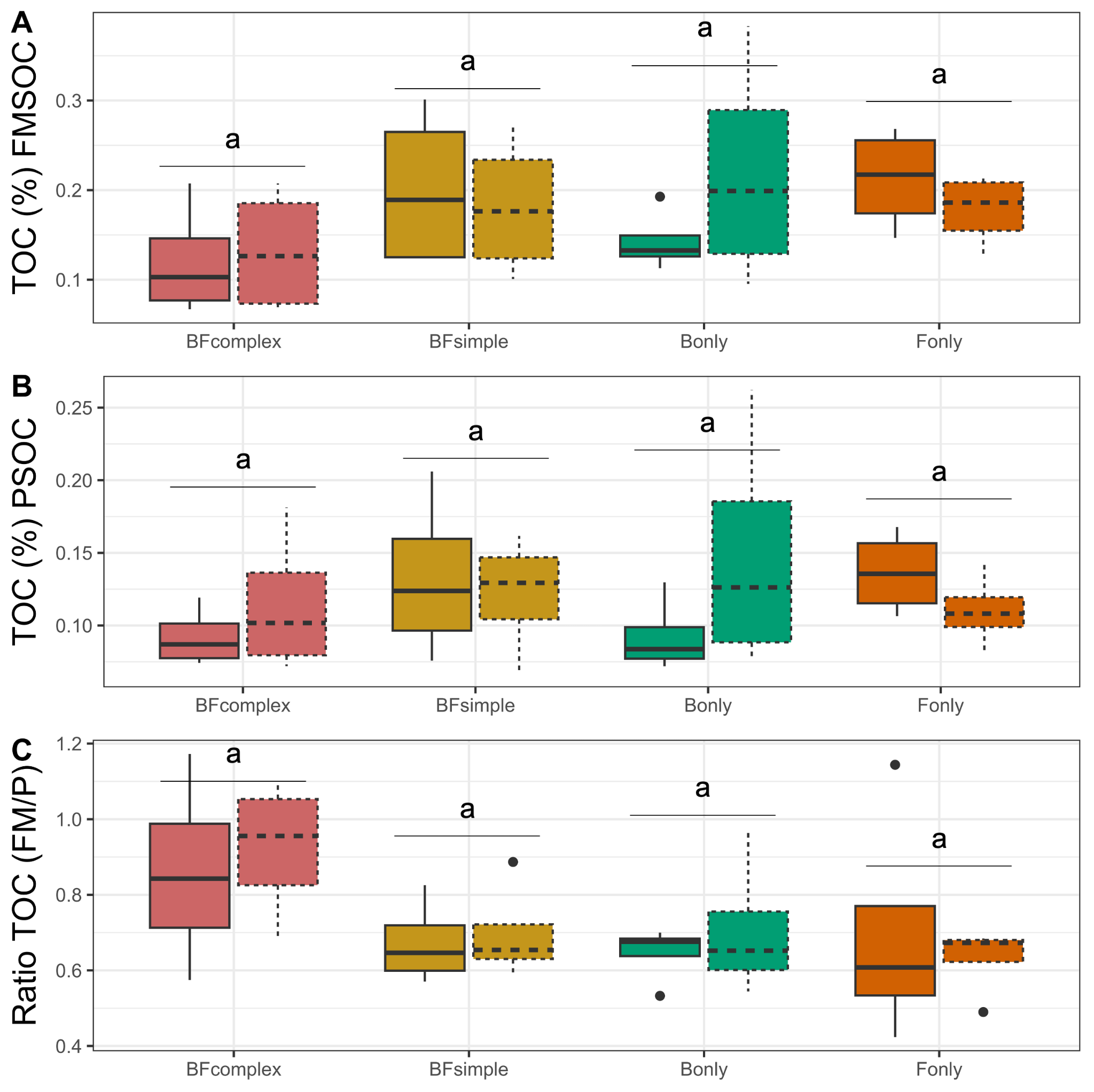  **Supplementary Figure 2. Total organic carbon (TOC) measured in these model soils.** TOC (%) measured at the end of the first incubation phase FMSOM (A), TOC (%) measured at the end of the second incubation phase PSOM (B) and the ratio between the TOC measured in the FMSOM and PSOM phases. Significant differences between treatments are indicated with different letters (anova followed by Tukey HSD test, P < 0.05) or significant t-test results (*P* < 0.05). |
| --- |

| 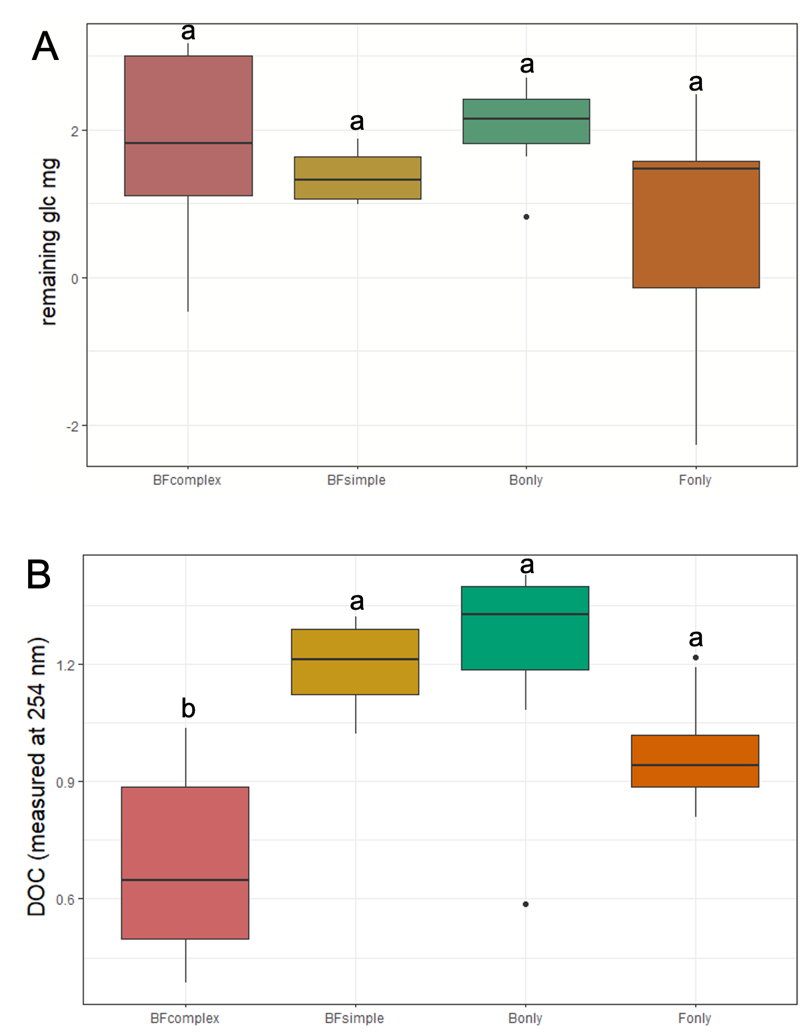 |
| --- |
| **Supplementary Figure 3. Estimates of organic carbon remaining in the soils at the end of FMSOM.** Estimated remaining glucose taking into account the cumulative respiration, necromass accumulated during the incubation and the microbial biomass measured at the end of FMSOC incubation (A). Absorbance measured at 254 nm as an estimate of how much dissolved organic carbon was present in the distinct soils. Significant differences between treatments are indicated with different letters (anova followed by Tukey HSD test, P < 0.05) or significant t-test results (*P* < 0.05). |


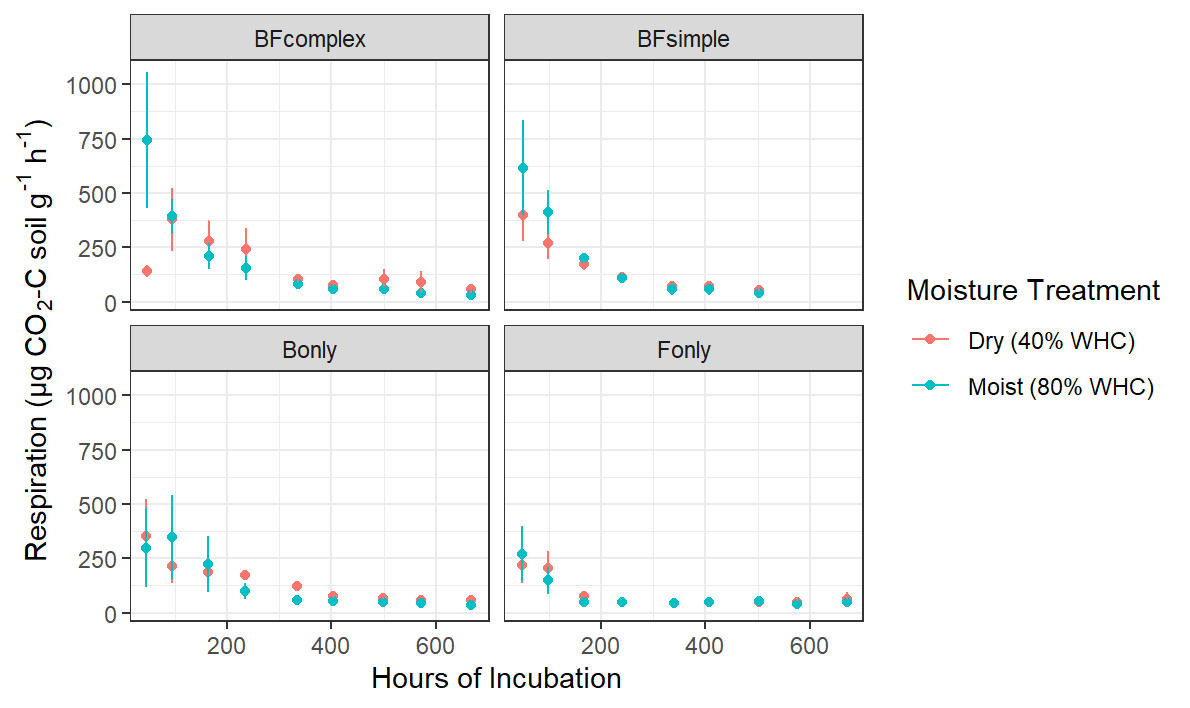


**Supplementary Figure 4.** Respiration measurements during the first phase of incubation for each treatments and moisture level.


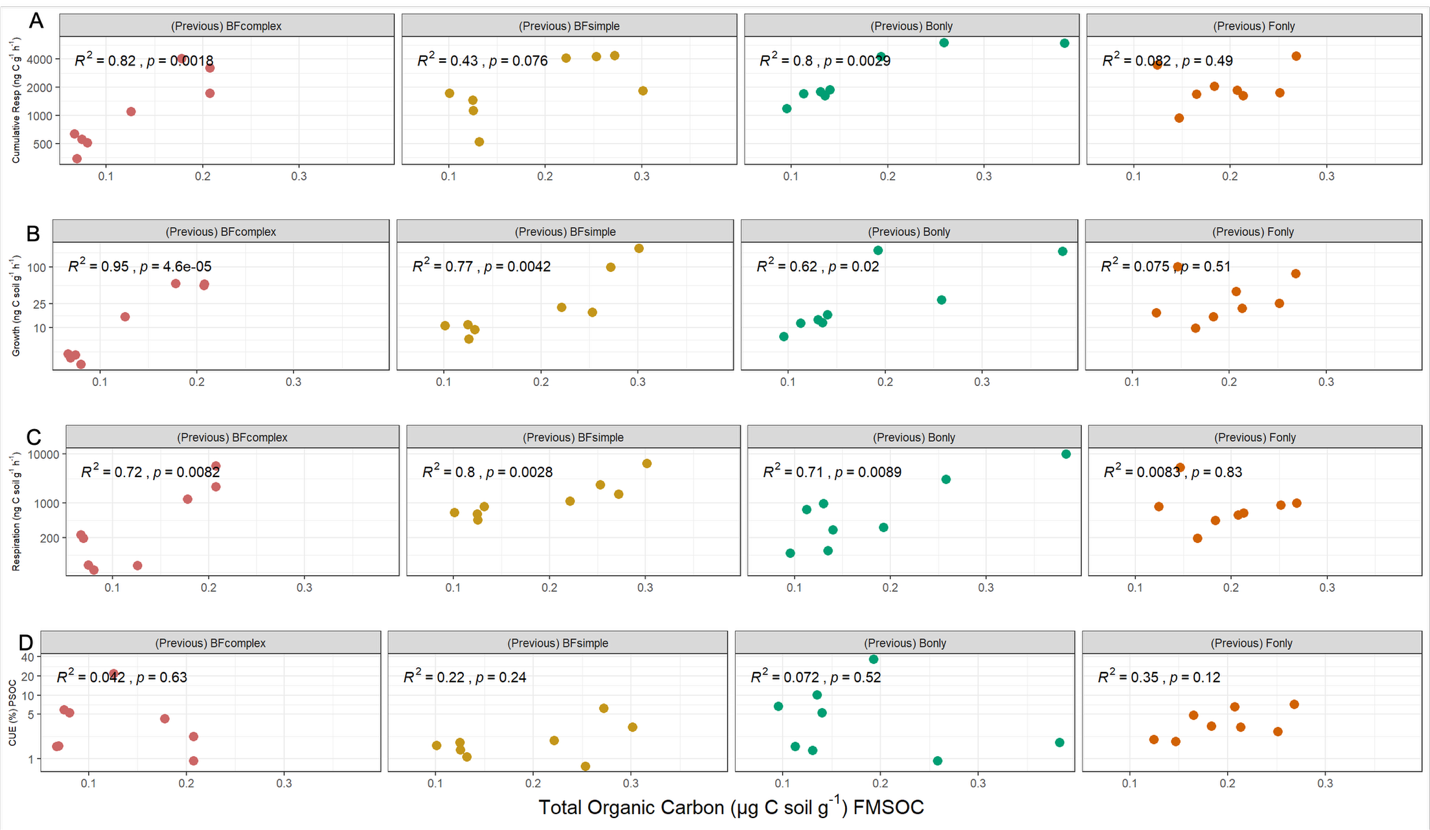


Supplementary Figure 5. Relationship between total organic carbon (TOC) measured at the end of FMSOM phase and microbial C-cycling processes measured during PSOM for each community type. A) Relationship between the microbial activity measured as cumulative respiration for two weeks during PSOM phase and the TOC. B) Relationship between growth measured at the end of PSOM and the TOC. C) Relationship between respiration measurement at the end of PSOM and the TOC. D) Relationship between CUE measured at the end of PSOM and the TOC.


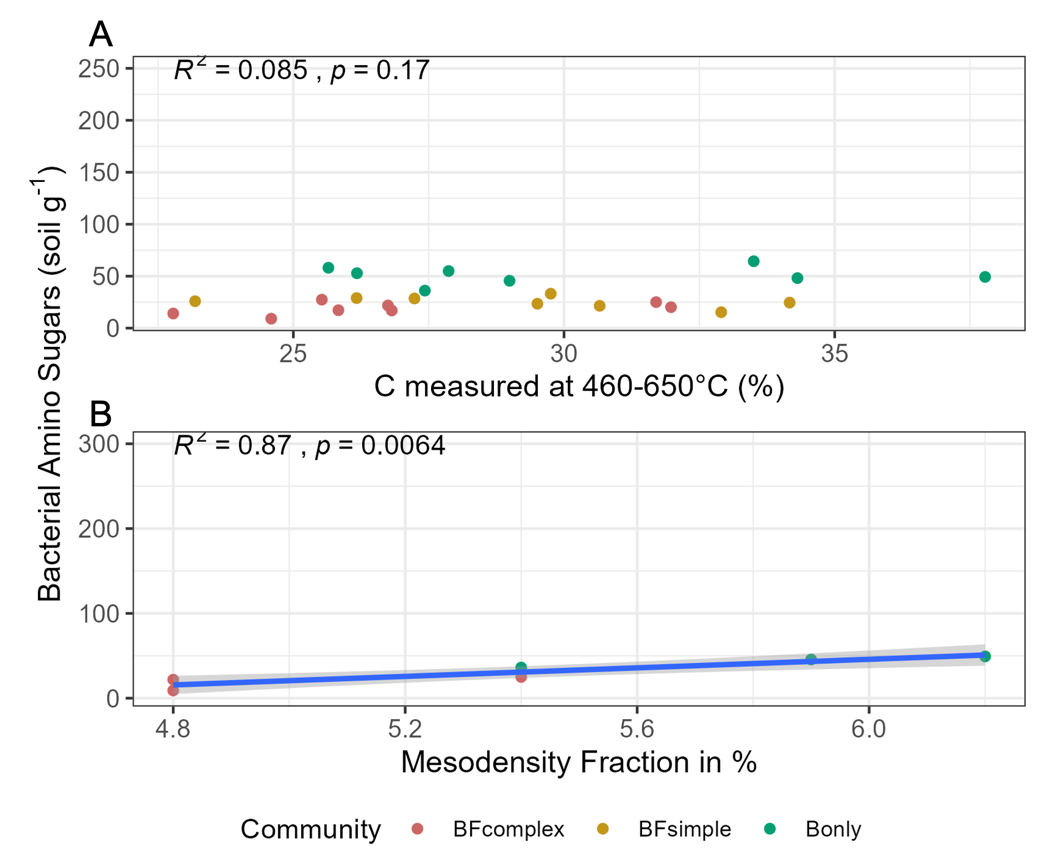


Supplementary Figure 6. Relationship between bacterial amino sugars and carbon captured between 460-650 °C during ramped thermal pyrolysis (A), relationship between and bacterial amino-sugar and the mass proportion of meso-density aggregate formed (B).


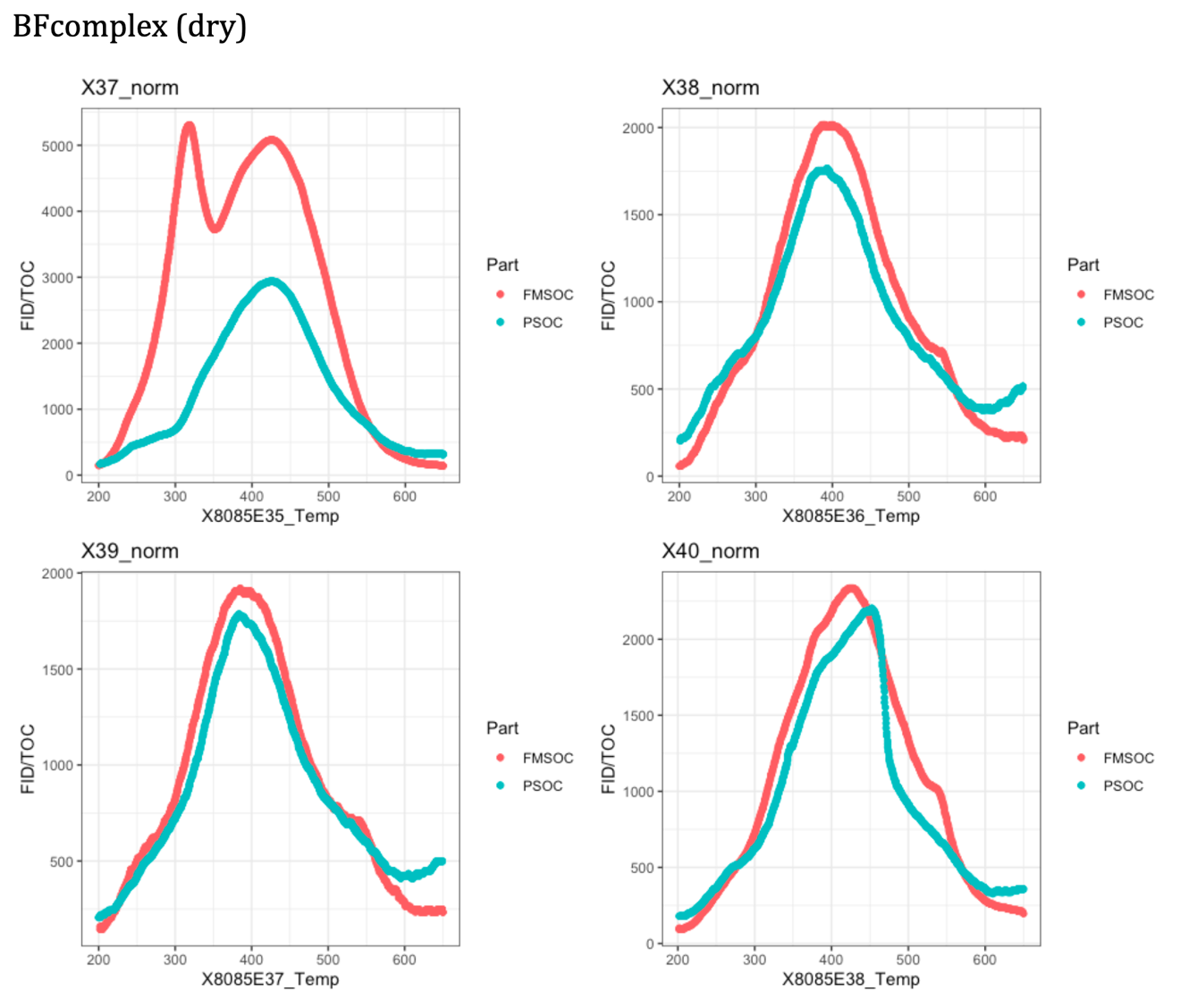


Supplementary Figure 7. Flame ionization detection (FID) signal normalized by total organic carbon (TOC), for the FMSOC and PSOC phases, for the four replicates of BF complex treatment at 40% water holding capacity (WHC).


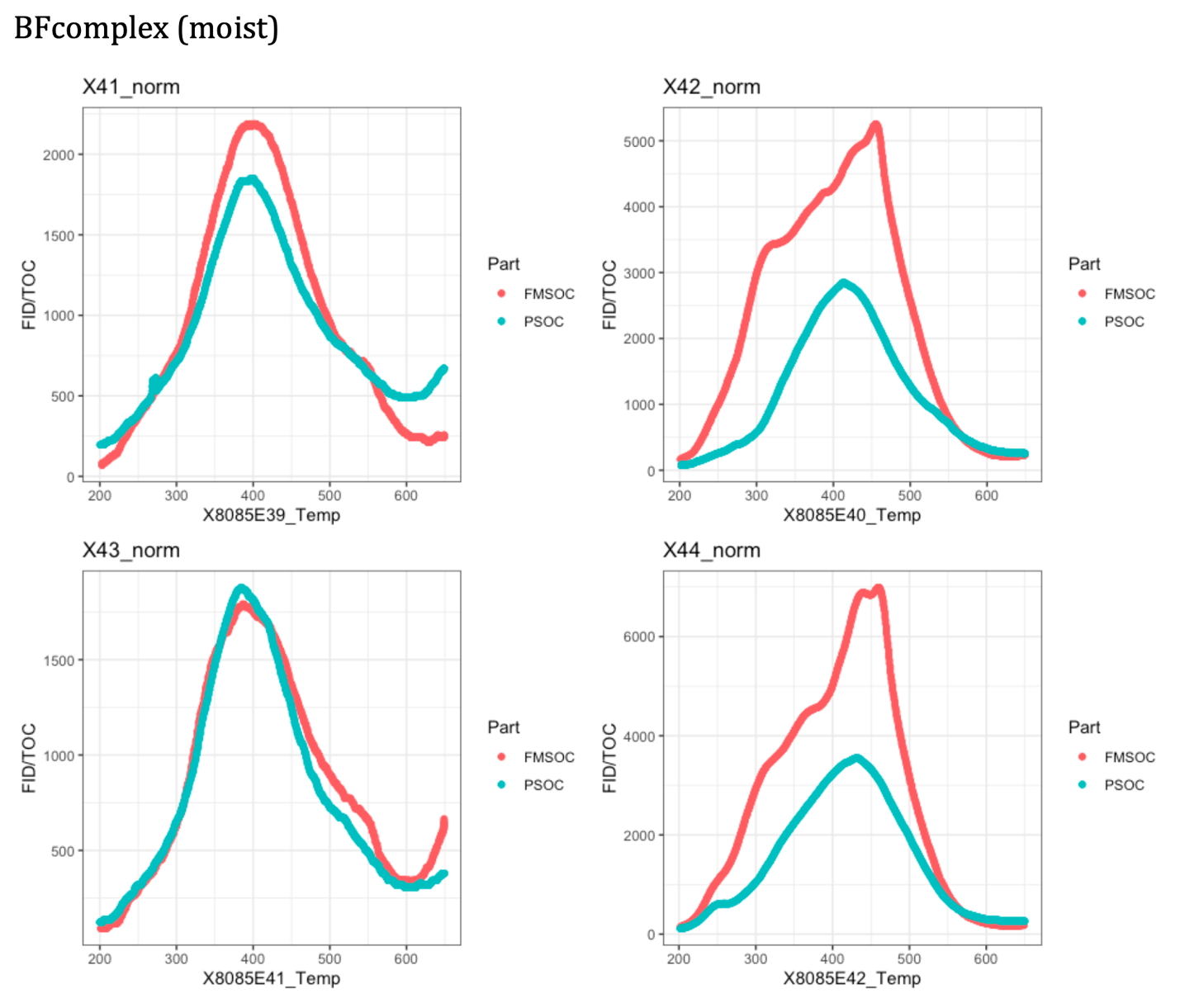


Supplementary Figure 8. Flame ionization detection (FID) signal normalized by total organic carbon (TOC), for the FMSOC and PSOC phases, for the four replicates of BF complex treatment at 80% water holding capacity (WHC).


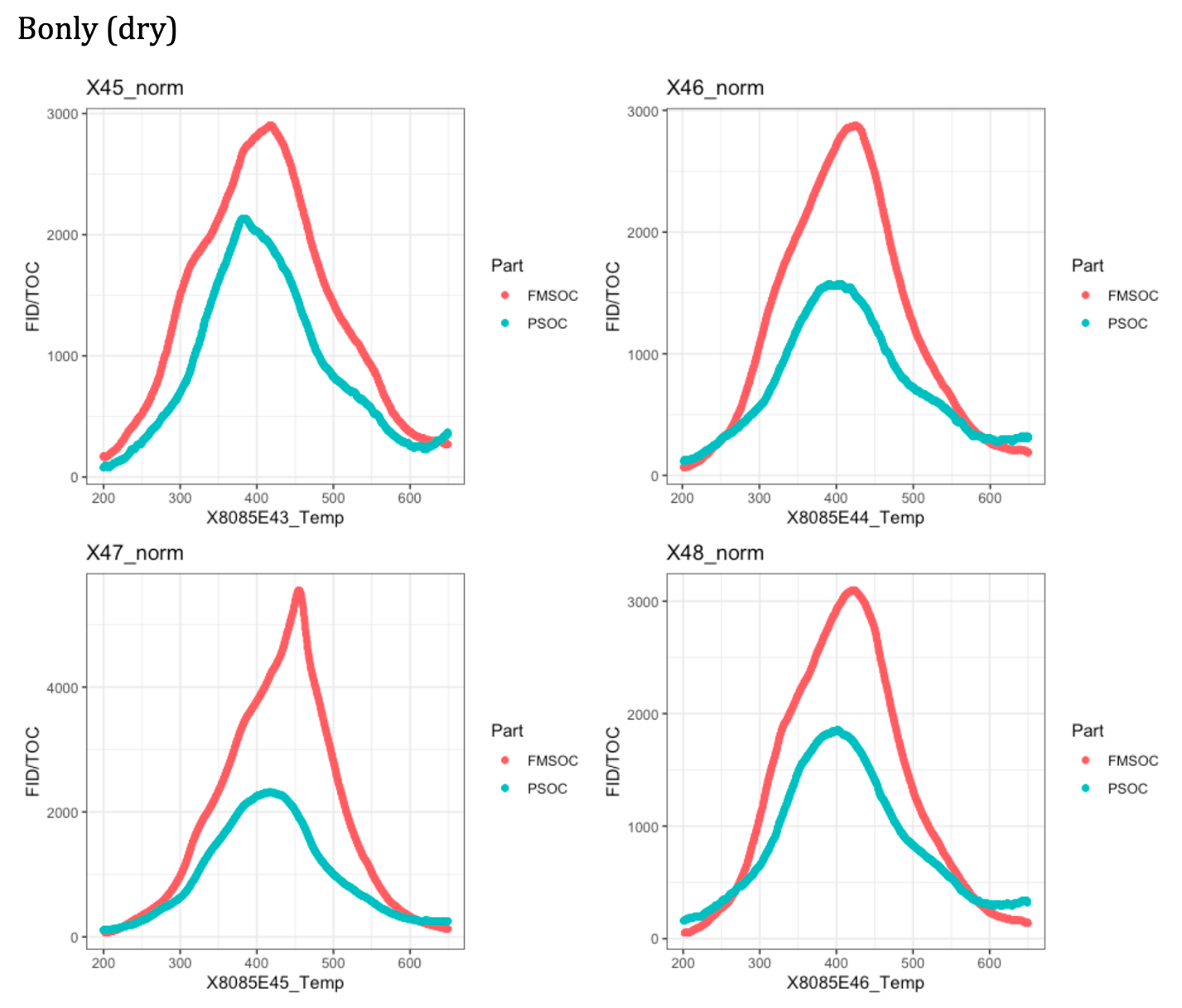


Supplementary Figure 9. Flame ionization detection (FID) signal normalized by total organic carbon (TOC), for the FMSOC and PSOC phases, for the four replicates of Bonly treatment at 40% water holding capacity (WHC).


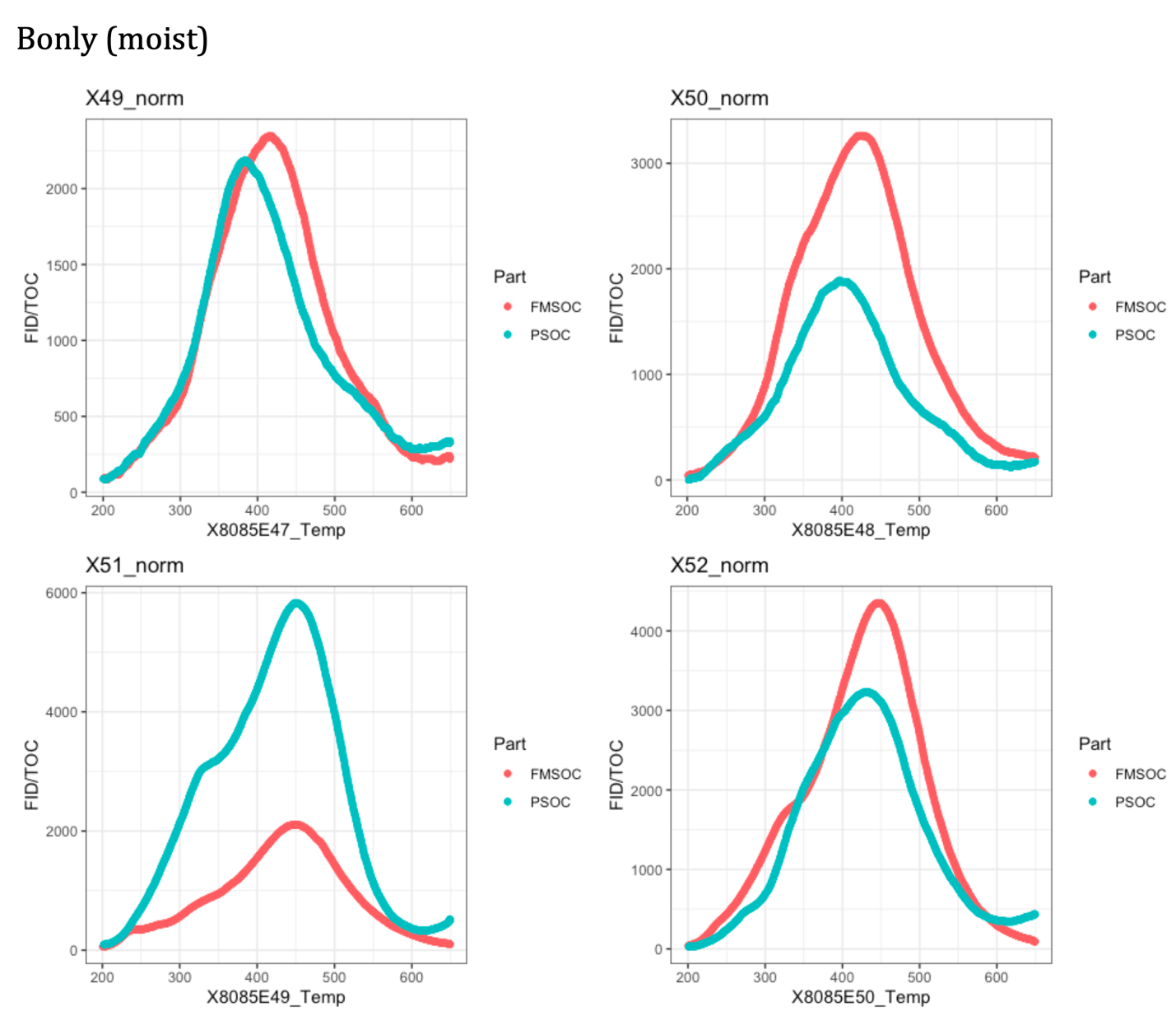


Supplementary Figure 10. Flame ionization detection (FID) signal normalized by total organic carbon (TOC), for the FMSOC and PSOC phases, for the four replicates of Bonly treatment at 80% water holding capacity (WHC).


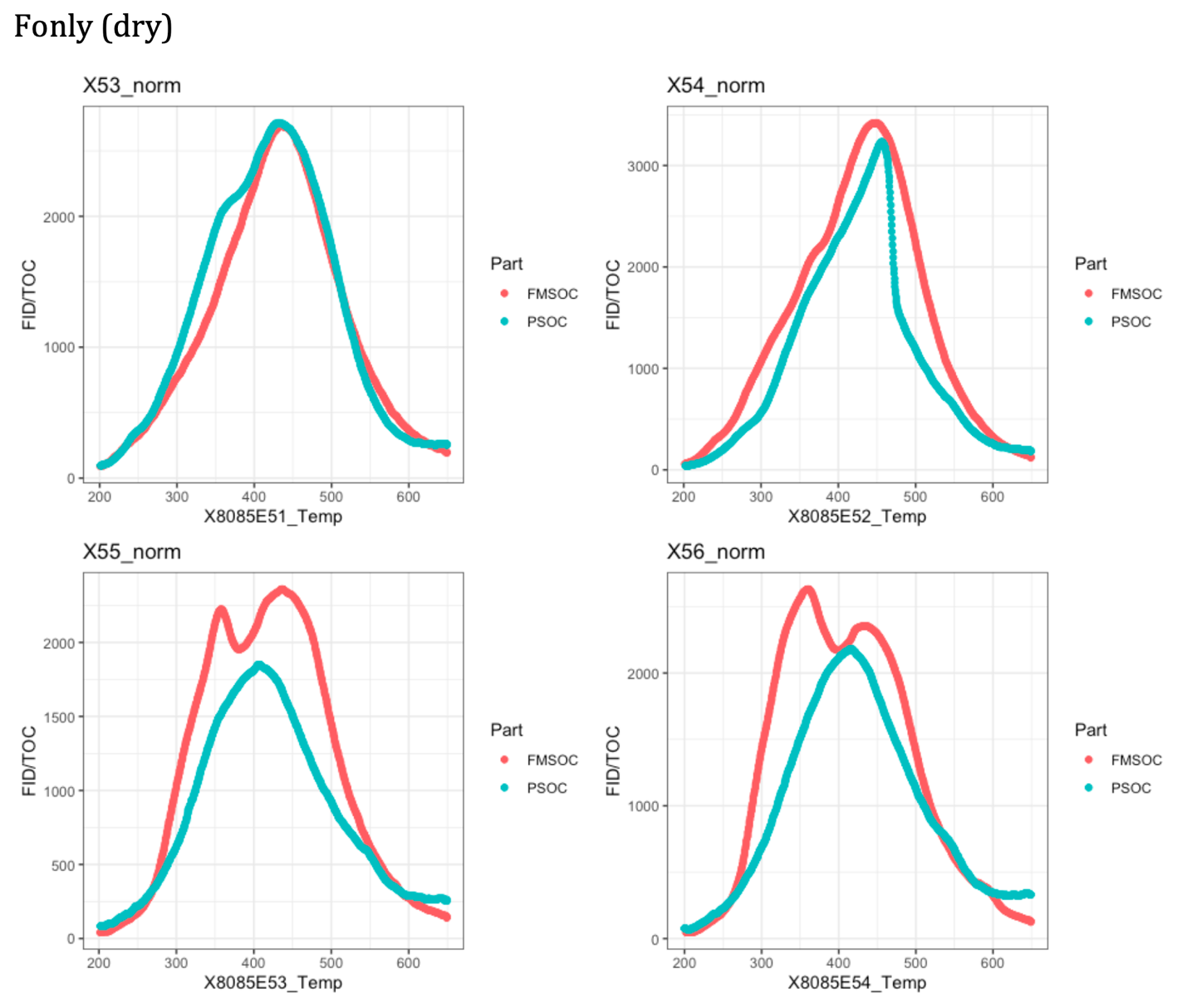


Supplementary Figure 11. Flame ionization detection (FID) signal normalized by total organic carbon (TOC), for the FMSOC and PSOC phases, for the four replicates of Fonly treatment at 40% water holding capacity (WHC).


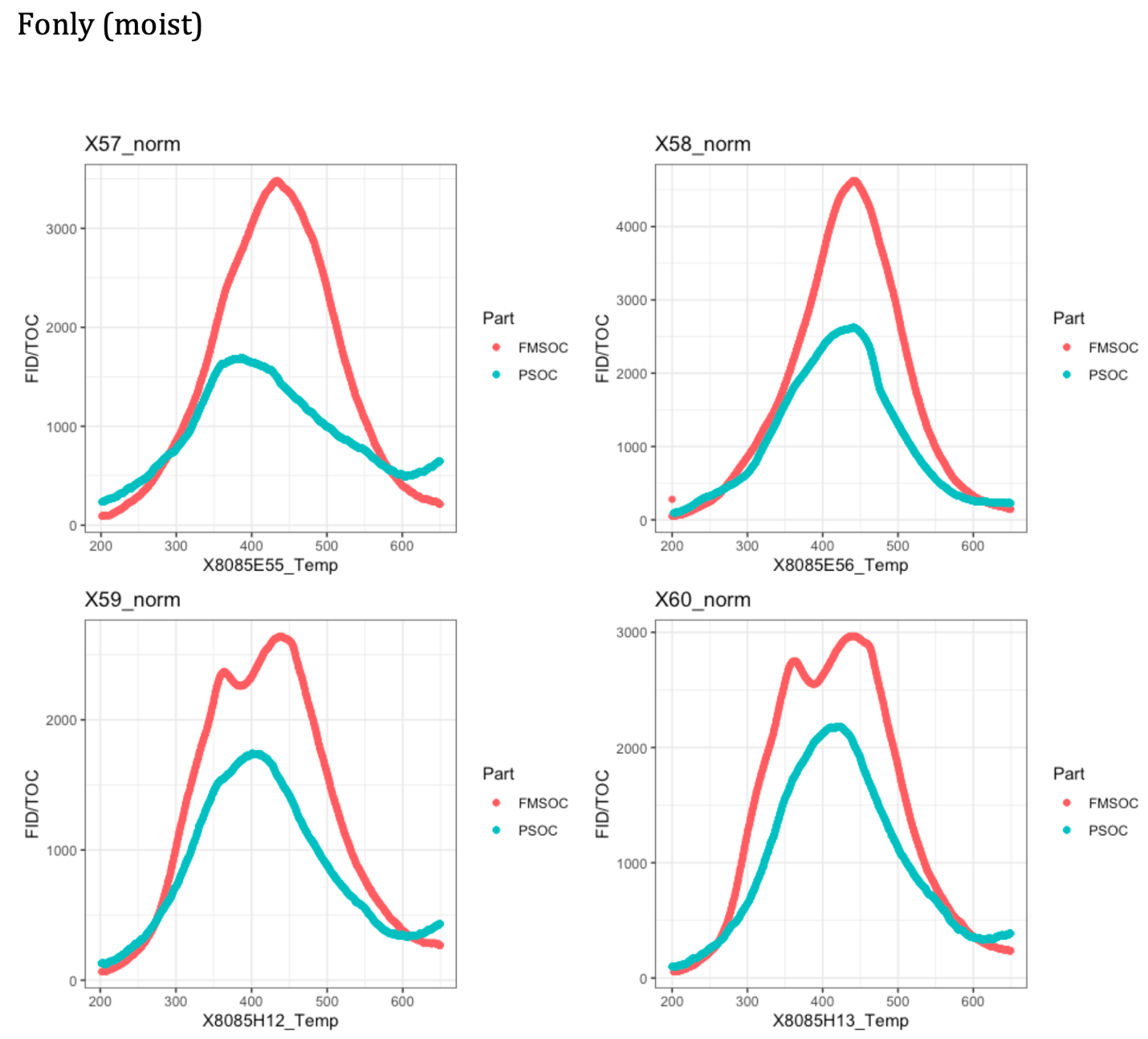


Supplementary Figure 12. Flame ionization detection (FID) signal normalized by total organic carbon (TOC), for the FMSOC and PSOC phases, for the four replicates of Fonly treatment at 80% water holding capacity (WHC).


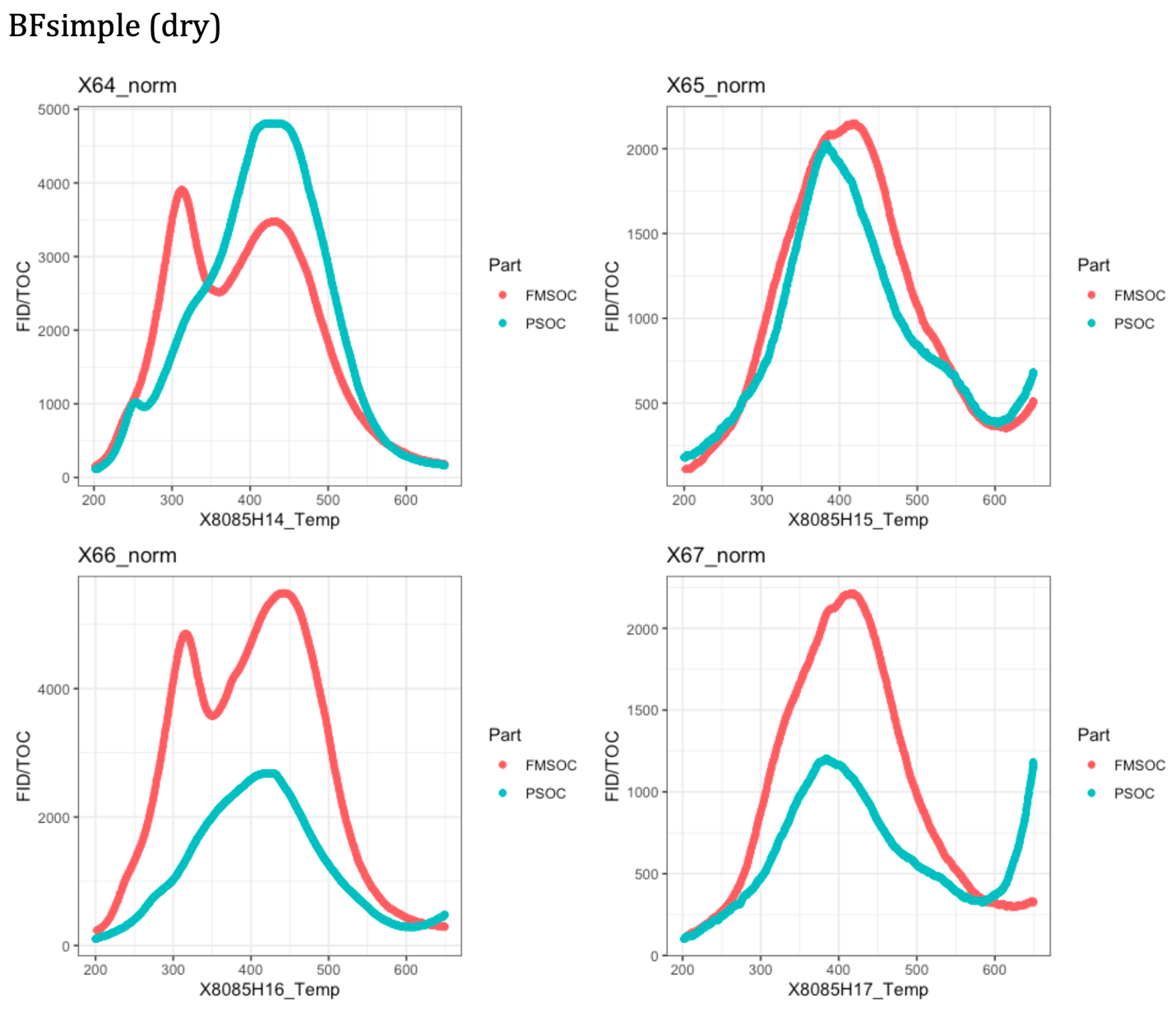


Supplementary Figure 13. Flame ionization detection (FID) signal normalized by total organic carbon (TOC), for the FMSOC and PSOC phases, for the four replicates of BFsimple treatment at 40% water holding capacity (WHC).


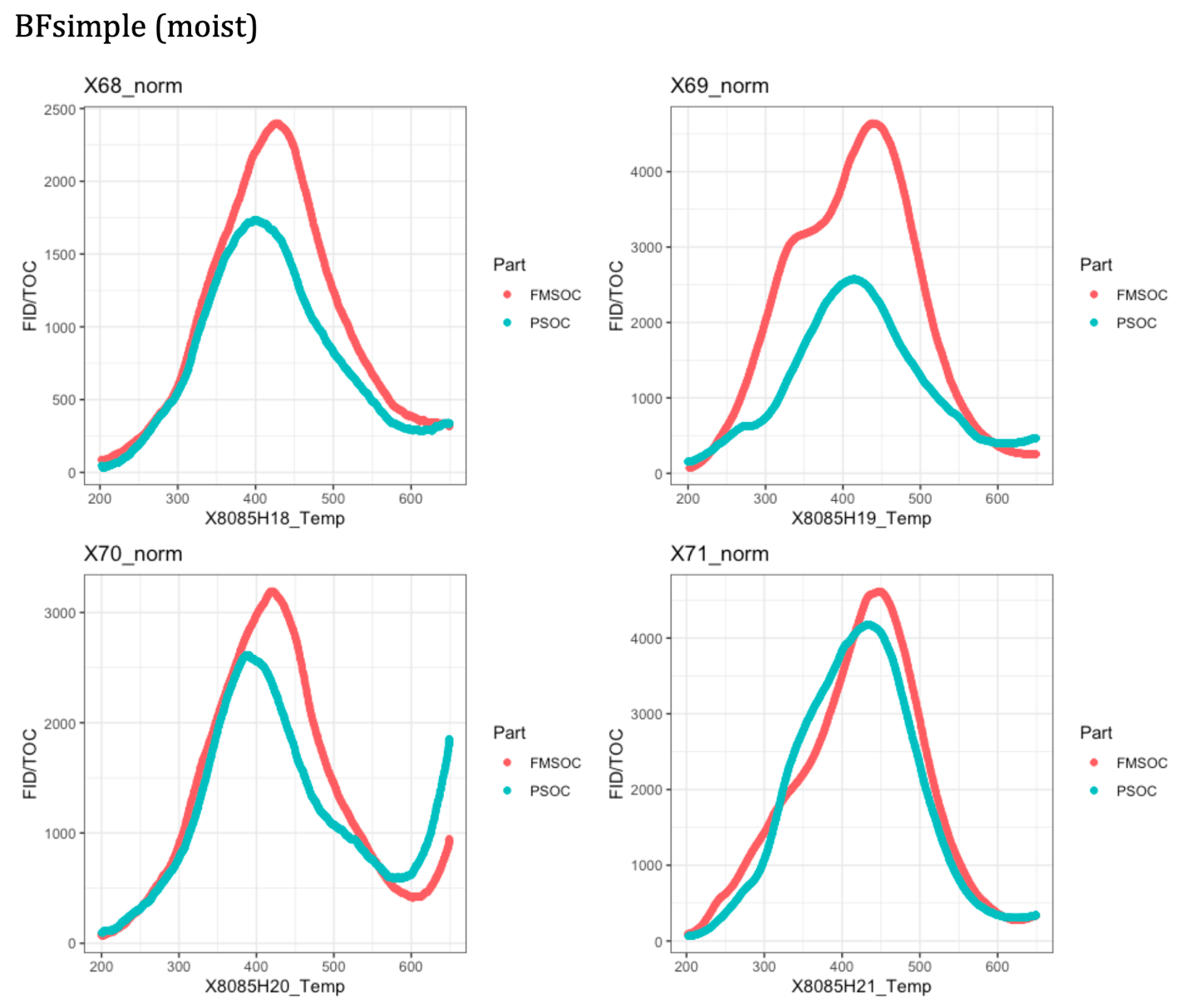


Supplementary Figure 14. Flame ionization detection (FID) signal normalized by total organic carbon (TOC), for the FMSOC and PSOC phases, for the four replicates of BFsimple treatment at 80% water holding capacity (WHC).
